# Satin bowerbird optimizer-neural network for approximating the capacity of CFST columns under compression

**DOI:** 10.1038/s41598-024-58756-7

**Published:** 2024-04-09

**Authors:** Yuzhen Liu, Yan Liang

**Affiliations:** 1 Bim School of Technology and Industry, Changchun Institute of Technology, Changchun, 130012 Jilin China; 2 Infrastructure Logistics Office, Jilin Engineering Normal University, Changchun, 130012 Jilin China

**Keywords:** Soft computing, Neural network, Metaheuristic algorithms, Concrete column; Satin bowerbird optimizer, Neuroscience, Mathematics and computing

## Abstract

Concrete-filled steel tube columns (CFSTCs) are important elements in the construction sector and predictive analysis of their behavior is essential. Recent works have revealed the potential of metaheuristic-assisted approximators for this purpose. The main idea of this paper, therefore, is to introduce a novel integrative model for appraising the axial compression capacity (*P*_*u*_) of CFSTCs. The proposed model represents an artificial neural network (ANN) supervised by satin bowerbird optimizer (SBO). In other words, this metaheuristic algorithm trains the ANN optimally to find the best contribution of input parameters to the *P*_*u*_. In this sense, column length and the compressive strength of concrete, as well as the characteristics of the steel tube (i.e., diameter, thickness, yield stress, and ultimate stress), are considered input data. The prediction results are compared to five ANNs supervised by backtracking search algorithm (BSA), earthworm optimization algorithm (EWA), social spider algorithm (SOSA), salp swarm algorithm (SSA), and wind-driven optimization. Evaluating various accuracy indicators showed that the proposed model surpassed all of them in both learning and reproducing the *P*_*u*_ pattern. The obtained values of mean absolute percentage error of the SBO-ANN was 2.3082% versus 4.3821%, 17.4724%, 15.7898%, 4.2317%, and 3.6884% for the BSA-ANN, EWA-ANN, SOSA-ANN, SSA-ANN and WDA-ANN, respectively. The higher accuracy of the SBO-ANN against several hybrid models from earlier literature was also deduced. Moreover, the outcomes of principal component analysis on the dataset showed that the yield stress, diameter, and ultimate stress of the steel tube are the three most important factors in* P*_*u*_ prediction. A predictive formula is finally derived from the optimized SBO-ANN by extracting and organizing the weights and biases of the ANN. Owing to the accurate estimation shown by this model, the derived formula can reliably predict the *P*_*u*_ of concrete-filled steel tube columns.

## Introduction

The world of engineering has witnessed continuous development of sophisticated algorithms and apparatus that lead to less complicated calculations and easier implementations of evaluative models^1–4^. For instance, many experimental efforts have used such developments to evaluate the quality of construction materials^5–8^. Focusing on civil and structural engineering, experts have benefited from novel approaches to analyze structural elements and construction materials such as concrete and steel^9–12^. Above many construction materials, concrete has been broadly used for various civil engineering projects^13–17^. Steel is another popular material that, owing to specific advantages, has received huge attention in generating structural elements^18,19^. In recent decades, engineers have suggested using composite structural elements to take advantage of both concrete and steel^20–22^. Becoming an effective construction material, numerous studies have been dedicated to assessing the capacity of composite structures. For instance, Shakouri Mahmoudabadi et al.^23^ investigated the behavior of concrete columns fortified with glass fiber-reinforced polymer bars subjected to eccentric loading using experimental and finite element analysis models. They observed that the loading capacity of the specimens declines as the eccentricity rises. As a particular type, concrete-filled steel tube column (CFSTC) is highly regarded in civil engineering works worldwide^24–26^. In this regard, many experimental and numerical approaches have been developed for exploring their behaviors^27–29^, and more particularly, the axial compression capacity (*P*_*u*_)^30,31^.

Due to the highly non-linear relationship between the mechanical parameters of construction material and influential characteristics, recent scientific efforts advice employing machine learning like artificial neural network (ANN)^32^, gradient tree boosting algorithm^33^, support vector regression (SVR)^34^, and adaptive neuro-fuzzy inference system (ANFIS)^35^ models for such purposes. These models are able to map and reproduce the intrinsic dependency of any output parameter on its corresponding inputs^36–38^. For example, Ghasemi and Naser^39^ could successfully use two explainable artificial intelligence techniques called XGBoost and random forest to predict the compressive strength of 3D concrete mixtures. These techniques also revealed the pivotal role of specimen age and fine aggregate quantity in the prediction task. As for the *P*_*u*_-related simulations, many scholars have benefited from these models to establish a firm predictive intelligence. Le^40^ could predict the bearing capacity of elliptical CFSTC subjected to axial load using ANFIS and present a graphical user interface for this purpose. Ahmadi et al.^41^ professed the applicability of ANN and also its superiority over experimental tools for the same objective. The suggested model could achieve correlation values of around 0.93 in the training and validation phases, and about 0.90 in the testing phase. A powerful ANN was optimized and used by Tran et al.^42,43^. This model, along with sensitivity analysis, investigated the effect of inputs and pointed out the steel tube diameter as the most efficient factor. Gene expression programming is another popular intelligent model that was hired by Nour and Güneyisi^44^ for evaluating the ultimate strength of CFSTC created from recycled aggregate concrete. Naser et al.^45^ presented another successful use of this algorithm.

More sophisticated efforts that sought optimal solutions resulted in designing capable search strategies for intricate problems^46–48^. These models are called metaheuristic techniques that simulate the problem in their specific environment and finally provide the optimum solution^49–51^. The pivotal objective of many studies has been showing the optimization competency of these algorithms^52–54^. A well-known application of metaheuristic is assisting conventional predictors toward a more reliable performance. Mai et al.^55^ proposed the combination of radial basis function (RBF) ANN with firefly algorithm (FFA), differential evolution (DE), and genetic algorithm (GA) for estimating the *P*_*u*_ of square CFSTC. A comparison showed that the RBF-FFA model can perform 28, 37, and 52% more accurately than RBF-GA, RBF-DE, and conventional ANN, respectively. Likewise, Ren et al.^56^ synthesized particle swarm optimization (PSO) and support vector machine for analyzing the ultimate bearing capacity of CFSTC. Due to the higher accuracy, the proposed model was preferred over theoretical and empirical techniques. Hanoon et al.^57^ trained an ANN with a PSO algorithm and achieved a good accuracy (coefficient of variation between 4.98% and 9.53%) in evaluating the flexural bending capacity of CFST beams. Ngo and Le^58^ incorporated SVR, which is a popular intelligent predictor, with grey wolf optimization (GWO) for analyzing the bearing capacity of CFSTCs. Due to the considerable accuracy improvements caused by the proposed model (from 10.3 to 87.9%), it was introduced as an effective tool for this purpose. Further similar applications of such algorithms can be found for invasive weed optimization (IWO)^59^, genetic algorithm (GA)^60^, and balancing composite motion optimization (BCMO)^61^.

From the above-discussed studies, it can be found that the combination of regular predictors with metaheuristic algorithms makes promising evaluative models for various concrete-related parameters^62,63^. On the other hand, the advent of new metaheuristic algorithms calls for extensive investigations into the suitability of the existing models. This study is therefore concerned with designing a novel integrative model based on ANN supervised by satin bowerbird optimizer (SBO)^64^ for estimating the *P*_*u*_ of CCFSTC. Moreover, to have a comparative approach, the performance of the SBO is compared to five other optimizers, namely backtracking search algorithm (BSA)^65^, earthworm optimization algorithm (EWA)^66^, social spider algorithm (SOSA)^67^, salp swarm algorithm (SSA)^68^, and wind-driven optimization (WDO)^69^ in the present study, as well as several methods in the previous literature. It is worth mentioning that the selected algorithms have not been earlier used for this purpose; and owing to the comparisons that will be performed among a large number of techniques, the findings of this research provide valuable insights into the literature of machine learning applications in estimating the *P*_*u*_ of CCFSTCs. The optimum configurations of the used models are discovered to predict the *P*_*u*_ from related geometrical and physical parameters. Two other outcomes of this study are (i) implementing statistical analysis on the *P*_*u*_ dataset to identify the most important parameters and (ii) a mathematical monolithic formula that can eliminate the need for computer-aided computations for calculating the *P*_*u*_.

In the following, the manuscript is organized as follows: Section “Materials and methods” describes the used material (i.e., data, algorithms, and accuracy criteria), Section “Results and discussion” presents the results along with relevant discussion about the findings, and Section “Conclusions” gives the conclusions.

## Materials and methods

### Data provision

**Figure 1:**
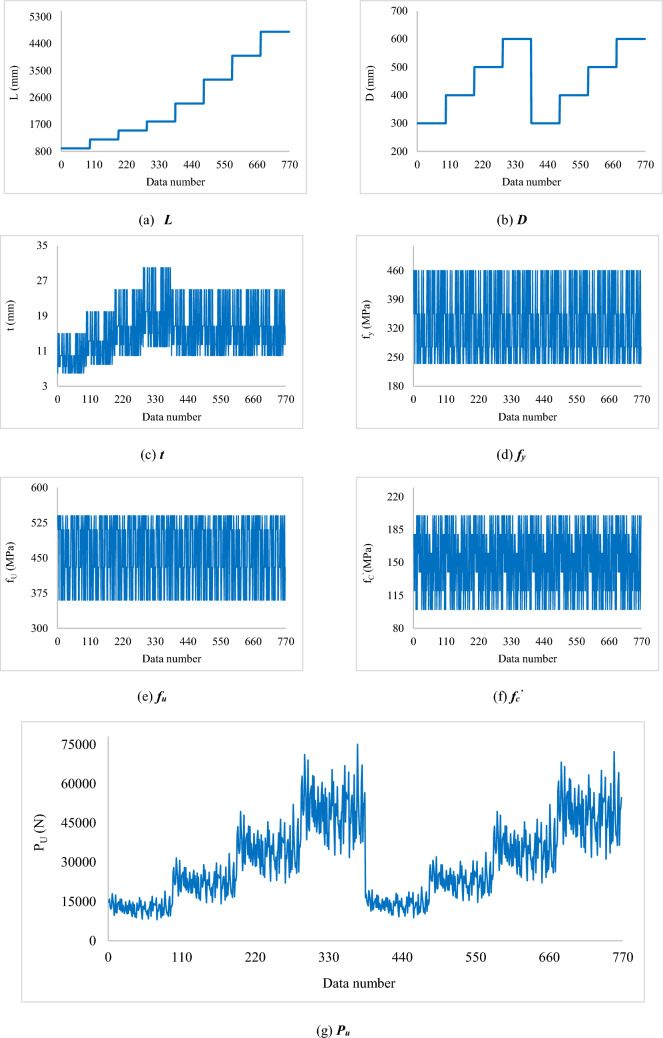
The individual behavior of the input and target parameters.

The CCFST data that is used for feeding the models of this research is taken from a previously done study by Tran et al.^70^. They analyzed the *P*_*u*_ of CCFSTC with ultra-high-strength concrete (UHSC) by finite element methods. A large dataset was produced that presents 768 *P*_*u*_ values versus some parameters that affect it. These parameters are called inputs (versus the *P*_*u*_ which is called target) that include column length (*L*), the diameter of steel tube (*D*), the thickness of steel tube (*t*), the yield stress of steel tube (*f*_*y*_), ultimate stress of steel tube (*f*_*u*_), and compressive strength of UHSC (*f*_*c*_^*’*^). Figure 1a–f show how these parameters change over the dataset. Likewise, Fig. 1g depicts the behavior of the *P*_*u*_. Also, Table 1 reports the statistical indicators of the dataset.

**Table 1:** Statistical description of the *P*_*u*_ and influential factors.

Factor	Descriptive indicator					
	Mean	Std. error	Std. deviation	Sample variance	Minimum	Maximum
*L* [mm]	2475.0	47.4	1313.1	1,724,119.9	900.0	4800.0
*D* [mm]	450.0	4.0	111.9	12,516.3	300.0	600.0
*t* [mm]	15.2	0.2	6.1	37.3	6.0	30.0
*f*_*y*_ [MPa]	331.3	3.1	86.0	7401.8	235.0	460.0
*f*_*u*_ [MPa]	460.0	2.5	70.4	4956.5	360.0	540.0
*f*_*c*_^*’*^ [MPa]	150.0	1.2	34.2	1168.2	100.0	200.0
*P*_*u*_ [N]	30,185.3	538.3	14,918.5	222,561,708.9	8016.3	75,051.6

Providing a sufficient number of samples to the machine learning models is of great importance in attaining a dependable analysis. The dataset consists of 768 records, which after permutation, were divided into two quite different parts with respect to the famous 80:20 ratio. The reason for permuting the dataset is to have samples from all parts of the dataset. These sub-datasets contain 614 and 154 samples which are used in the training and testing processes, respectively. In the training phase, the model explores the dependence of the *P*_*u*_ on the whole inputs and generates a pattern accordingly. Then, it applies the pattern to the smaller dataset to see how accurately the model can predict new *P*_*u*_s.

### The SBO

Based on the courtship and copulation of a so-called bird “satin bowerbird”, Moosavi and Bardsiri^64^ proposed a new optimization of the ANFIS called SBO. Up to now, many scholars have chosen this algorithm for their optimization purposes^71,72^. Moayedi and Mosavi^73^, for example, created a powerful hybrid of ANN using the SBO applied to electrical load prediction. The algorithm draws on six major steps: (a) random generation of the population, probability calculation (for each individual), elitism, spotting changes in the positions, mutation, and finally synthesizing old and new populations^64^.

In a more clear description, after creating a random population, the position of each bird is presented by a K-dimensional vector. Next, the algorithm calculates a probability value based on Eq. 1 that stands for the attractiveness of the birds.

1 $${P}_{i}= \frac{{fit}_{i}}{\sum_{n = 1}^{K}{fit}_{n}},$$

in which ${fit}_{i}$ gives the fitness of the *i*th bird obtained from the below equation:

2 $${Fit}_{i}= \left\{\begin{array}{c}\frac{1}{1+f({X}_{k})}\quad  f({X}_{k})\ge 0\\ 1+\left|f({X}_{k})\right| \quad f\left({X}_{k}\right)<0\end{array}\right.,$$

where $f({X}_{k})$ stands for the cost function of the bower k. These values are compared in the elitism step to select the best-fitted member. In this regard, the higher the fitness is, the better the solution is.

Equation 3 expresses the adjustment of other bowerbirds’ positions throughout iterative efforts.

3 $${X}_{ij}^{new}={X}_{ij}^{old}+ {\lambda }_{j}\left[\left(\frac{{X}_{kj}+ {X}_{best,j}}{2}\right)- {X}_{ij}^{old}\right],$$

in which ${\lambda }_{j}$ is step length indicator, ${X}_{ij}$ stands for the element j in the position vector of the bowerbird i (likewise ${X}_{best,j}$ denotes this element in the position vector of the best bowerbird), noting that j is obtained from the roulette wheel technique. In this algorithm, more experienced bowerbirds may eliminate weaker ones in the courtship competition. It leads to a mutation process which can be expressed by the below relationship^74,75^.

4 $${X}_{ij}^{new}\sim N {(X}_{ij}^{old}, {\sigma }^{2}), \sigma = z \times \left({Var}_{max}- {Var}_{min}\right),$$

where the maximum and minimum values of variables are respectively denoted by ${Var}_{max}$ and ${Var}_{min}$ and the difference between them is shown by z. Lastly, the former population is combined with the new ones at the end of each cycle. The whole population is then evaluated and sorted with respect to the fitness values and those with the lowest cost are preserved. This process continues iteratively until a computational goal is satisfied^76^

### The benchmarks

Toward a comparative assessment of the proposed model, five different metaheuristic methods, namely BSA, EWA, SOSA, SSA, and WDO are used in this work. The same duty of the SBO (i.e., training the ANN) is assigned to these algorithms. While each algorithm simulates the problem based on a specific strategy, they are all known as population-based techniques. It means that each algorithm hires a population of search agents (e.g., earthworms in the EWA) to seek the optimum solution in the problem space. After designating proper parameters (e.g., the population size), relevant physical/natural rules are applied to provide optimal training for the ANN. Another similarity among these algorithms is that they need to be implemented for a large number of iterations (e.g., 1000) to minimize the cost function properly (will be explained in Section “Network optimization (training)”). The overall description of these strategies is presented in Table 2 and further methodological details can be found in studies given in the last column.

**Table 2:** Description of the used benchmark algorithms.

Name	Developer(s)	Year	Inspiration/Strategy	Examples of applications
BSA	Civicioglu^65^	2013	Backtracking search method (Dividing the problem into five steps (i) initialization, (ii) selection-I, (iii) mutation, (iv) crossover, and (v) selection-II) in virtual search space)	^77,78^
EWA	Wang et al.^66^	2018	Social behavior of earthworms (Burrowing action and two reproduction stages of earthworm along with Cauchy mutation)	^79,80^
SOSA	James and Li^67^	2015	Social spider behavior (Emulating male and female social spiders based on the regulations of cooperative colonies)	^81,82^
SSA	Mirjalili et al.^68^	2017	Swarming behavior of salp in chain movements (following the leaders toward food sources)	^83,84^
WDO	Bayraktar et al.^69^	2010	Wind and air parcel interaction (optimizing the air parcel by taking into consideration the effect of gravity, pressure gradient, Coriolis, and frictional forces W.R.T. Newton's second law plus ideal gas state equation)	^85,86^

### Accuracy assessment criteria

There are different indicators to assess the accuracy of predictive models. Each one follows a specific formula comparing the predicted and expected values of the simulated parameter. In this work, four famous ones, namely the RMSE, mean absolute error (MAE), mean absolute percentage error (MAPE), and Pearson correlation coefficient (R) are used. The first three indicators deal with the error of prediction, while R indicates the goodness of fit in a regression chart. The formulation of these indicators is defined as follows:

5 $$ RMSE = \sqrt {\frac{1}{N}\sum\limits_{i = 1}^{N} {[(P_{{ui_{observed} }} - P_{{ui_{estimated} }} )]}^{2} } , $$

6 $$ MAE = \frac{1}{N}\sum\limits_{i = 1}^{N} {|P_{{ui_{observed} }} - P_{{ui_{estimated} }} |} , $$

7 $$ MAPE = \frac{1}{N}\sum\limits_{i = 1}^{N} {|\frac{{P_{{ui_{observed} }} - P_{{ui_{estimated} }} }}{{P_{{ui_{observed} }} }}|} \times 100, $$

8 $$ R = \frac{{\sum\limits_{i = 1}^{N} {(P_{{ui_{estimated} }} - {\bar{P}_{u\,estimated} } )(P_{{ui_{observed} }} - {\bar{P}_{u\,observed} } )} }}{{\sqrt {\sum\limits_{i = 1}^{N} {(P_{{ui_{estimated} }} - {\bar{P}_{u\,estimated} } )^{2} } } \sqrt {\sum\limits_{i = 1}^{N} {(P_{{ui_{observed} }} - {\bar{P}_{u\,observed} } )^{2} } } }}, $$

where* N* represents the number of data. Also,${P}_{u {i}_{observed}}$ and ${P}_{u {i}_{estimated}}$ stand for the *i*th observed and estimated values of *P*_*u*_ (with averages of ${\overline{P} }_{u\,observed}$ and ${\overline{P} }_{u\,estimated}$), respectively.

## Results and discussion

### Network optimization (training)

The role of metaheuristic algorithms in combination with an ANN was explained in the previous sections. By unsupervised optimization, they achieve the optimal parameters (biases and weights) for the given ANN. Determining the structure of the ANN is a prerequisite of this process. The number of processors (i.e., neurons) in the hidden layers is an important variable. In this work, this variable is determined based on the previous experience of the authors supported by a trial-and-error test for the values. It was revealed that among 15 tested values (i.e., 1, 2, …, 15), five neurons build the most accurate network. So, given the number of inputs (i.e., six) and the single output, the ANN takes the format of 6 × 5 × 1.

**Figure 2:**
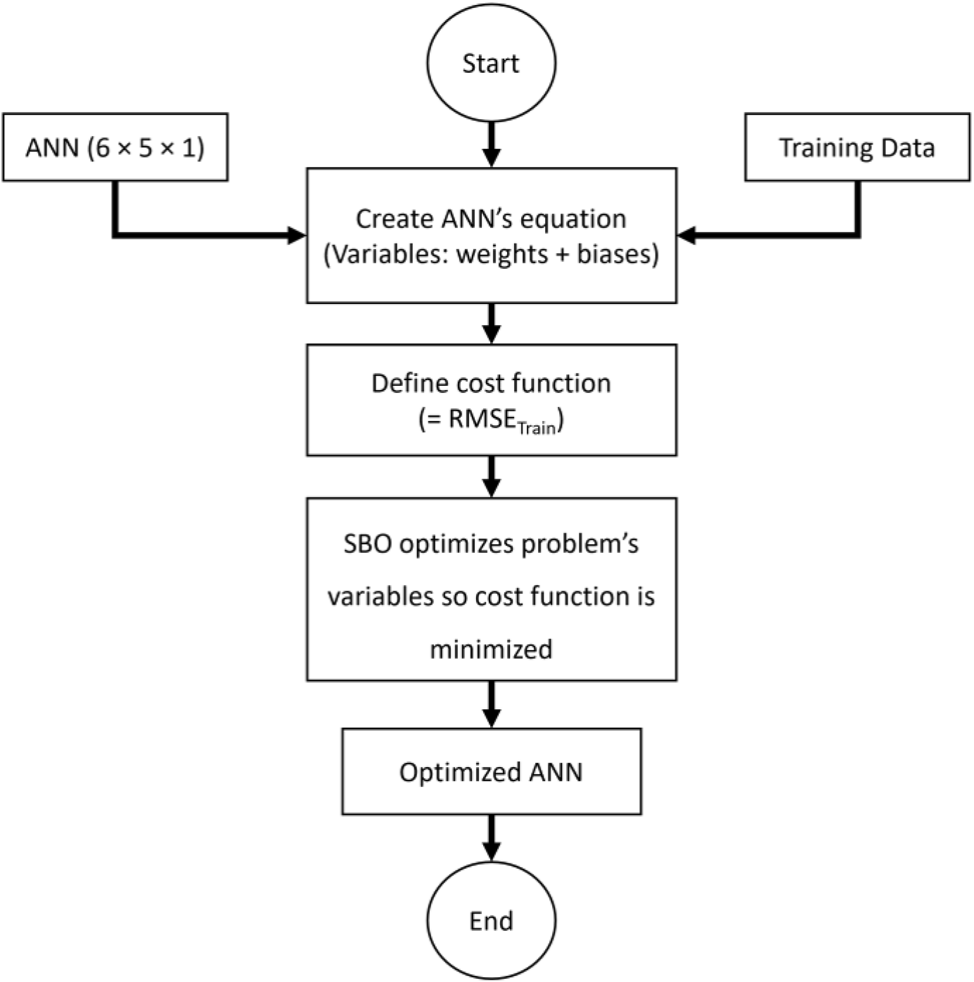
Flowchart of the optimization procedure.

The SBO algorithm was combined with the mentioned ANN to create the SBO-ANN hybrid. As illustrated in Fig. 2, this process has the following steps:

1. The selected ANN model is fed by the training dataset,
2. The mathematical representation of the ANN is created (will be explained in Section “An explicit formula”). The variables of this equation are the weights and biases of the ANN which must be tuned,
3. Training RMSE is designated as the objective function,
4. The mathematical ANN is exposed to the SBO algorithm as its optimization problem and the SBO tries to minimize this function so it achieves a lower RMSE (i.e., better training). This process is considered the main optimization step which is carried out by trying to improve the problem variables (i.e., weights and biases) in every iteration of the SBO.

**Figure 3:**
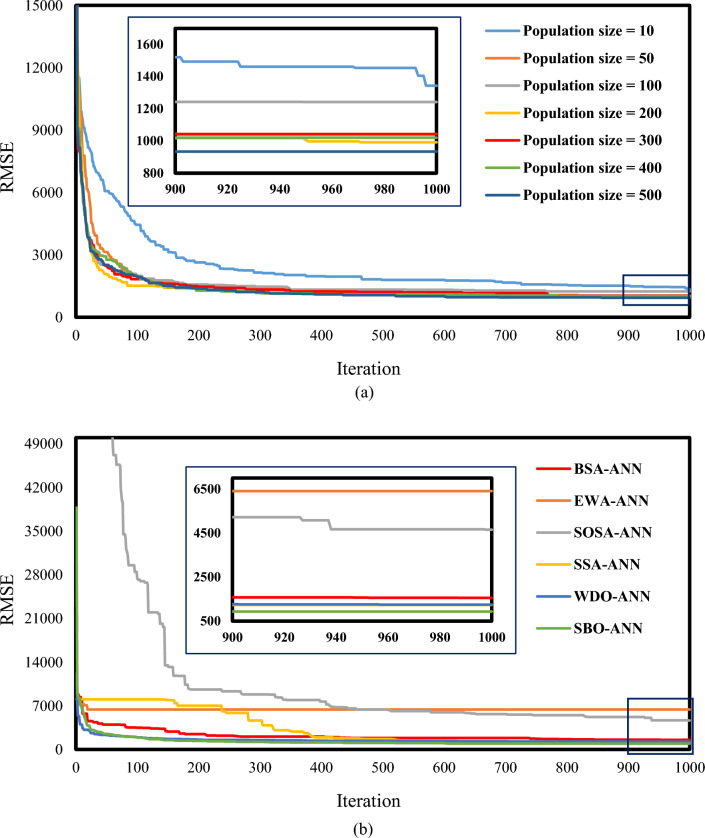
Convergence curves of (**a**) all tested SBO-ANNs and (**b**) the selected configurations of all used models.

A significant parameter of such optimization techniques is the size of the population (SoP). A well-accepted way to find a suitable SoP is by testing a wide range of them^87^. Figure 3a shows the convergence of the tested SBO-ANNs. According to this figure, all curves reach a relatively steady situation after one thousand iterations. Meanwhile, the training RMSE of each iteration gives the objective function (the *y*-axis). This figure also says that the lowest error is obtained for the SoP = 500. Thus, the results of this configuration will be considered for the SBO-ANN performance assessment.

The above efforts were executed for the benchmark algorithms (i.e., BSA, EWA, SOSA, SSA, and WDO) as well. In Fig. 3b, the convergence curves of all models are gathered and compared. Note that, the curves of the BSA-ANN, EWA-ANN, SOSA-ANN, SSA-ANN, and WDO-ANN belong to the SoPs of 400, 200, 200, 400, and 400, respectively. As is seen, there is a distinction between the final RMSE of the EWA-ANN and SOSA-ANN with others. Also, the RMSE of the SBO-ANN is below the benchmarks.

Knowing that optimization algorithms have a stochastic behavior, multiple runs are performed for each of the above conditions to ensure the repeatability of the results. Figure 3b, the RMSEs corresponding the initial solutions of the BSA, EWA, SOSA, SSA, WDO, and SBO were 8813.5833, 10,156.1479, 186,630.4071, 8056.0601, 12,194.4660, and 38,763.7112 which were minimized by these algorithms down to 1554.9111, 6408.0760, 4653.5890, 1233.5169, 1247.4574, 934.1530, respectively. These reductions show a nice optimization competency for all used algorithms concerning the problem at hand.

**Figure 4:**
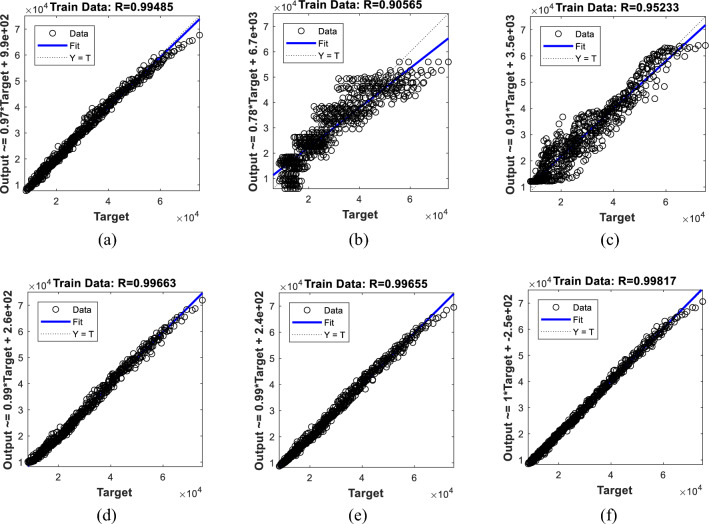
The regression-based evaluation of the training results obtained by the (**a**) BSA-ANN, (**b**) EWA-ANN, (**c**) SOSA-ANN, (**d**) SSA-ANN, (**e**) WDA-ANN, and (**f**) SBO-ANN.

These results show that the efforts of the SBO algorithm have been more productive relative to other algorithms. This superiority is professed by higher accuracy of training (i.e., lower error). To prove this, the outputs of the training data are compared to the observed *P*_*u*_s. Figure 4 illustrates this comparison in the form of regression charts. At a glance, the prediction of all six models is in very good agreement with expectations. However, the points of the BSA-ANN, SSA-ANN, and WDO-ANN are more aggregated than EWA-ANN and SOSA-ANN. The R values are obtained 0.99485, 0.90565, 0.95233, 0.99663, and 0.99655 for the BSA-ANN, EWA-ANN, SOSA-ANN, SSA-ANN, and WDO-ANN, respectively. As for the SBO-ANN, with the R-value of 0.99817, it outperformed all mentioned models.

The above comparison is indicated by other accuracy indicators, too. The RMSEs of the BSA-ANN, EWA-ANN, SOSA-ANN, SSA-ANN, WDO-ANN, and SBO-ANN were 1554.91, 6408.07, 4653.58, 1233.51, 1247.45, and 934.15, respectively (Fig. 4b). These values reflect the high quality of training carried out by the metaheuristic algorithms. The MAEs and the corresponding MAPEs were 1137.59 and 4.1591%, 5056.13 and 19.9943%, 3652.30 and 16.0975%, 965.20 and 3.7931%, 947.07 and 3.4434%, and 669.75 and 2.5060%. As these values imply, the training process is associated with tolerable and small errors. A low level of error means that the algorithms have nicely understood the neural relationship (between the *P*_*u*_ of CCFSTC and the *L*, *D*, *t*, *f*_*y*_, *f*_*u*_, and *f*_*c*_^*’*^) and have tuned the network parameters accordingly.

### Testing performance

As explained, the networks were initially derived from the information of 154 CCFSTCs in the training phase. This data was used to assess the efficiency of the models in dealing with unseen column conditions. In this process, when metaheuristic algorithms provide a calculation pattern for the ANNs, it should be demonstrated that this pattern can be applied to new problems.

**Figure 5:**
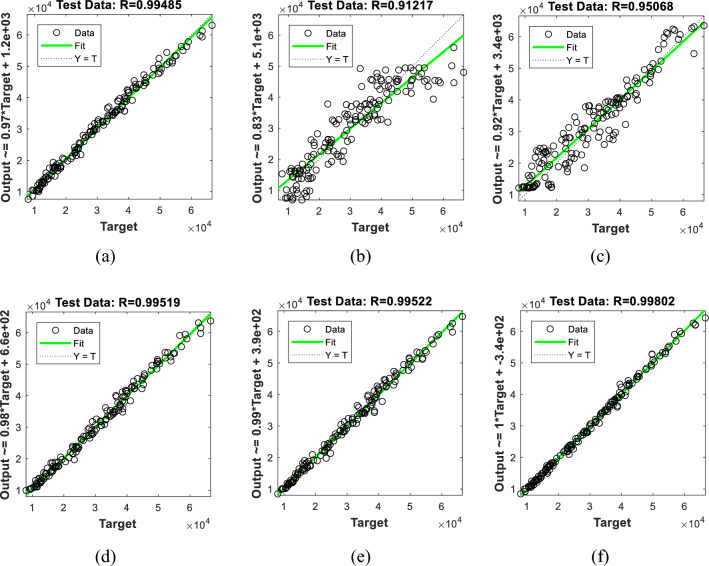
The regression-based evaluation of the testing results obtained by the (**a**) BSA-ANN, (**b**) EWA-ANN, (**c**) SOSA-ANN, (**d**) SSA-ANN, (**e**) WDA-ANN, and (**f**) SBO-ANN.

Figure 5 shows the regression charts of the testing data. Based on the R values of 0.99485, 0.91217, 0.95068, 0.99519, 0.99522, and 0.99802, all testing products show an excellent (> 91%) goodness-of-fit. Similar to Fig. 4, the points of the EWA-ANN and SOSA-ANN are more scattered compared to other models.

**Figure 6:**
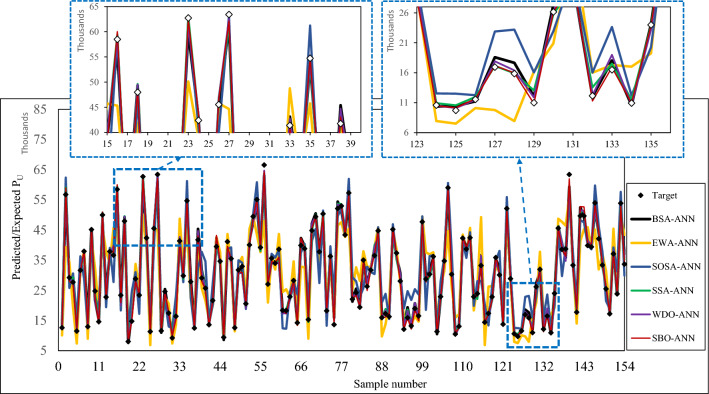
Comparison between the observed *P*_*u*_s and predicted patterns.

For further evaluation, Fig. 6 depicts the difference between the observed *P*_*u*_s and the pattern predicted by each model. The overall trend of the points is nicely estimated by all lines. No significant misleading has occurred and it shows that the neural-metaheuristic models can bear abrupt changes. Thus, the used models are competent enough to predict the *P*_*u*_ by taking the inputs. However, in compliance with previous results, the lines pertaining to the BSA-ANN, SSA-ANN, WDO-ANN, and SBO-ANN show a higher consistency with the observed values. Also, the magnified sections indicate that the smallest underestimating and overestimating cases (i.e., errors) are observed for the SBO-ANN line. Moreover, the RMSEs of 1507.82, 5906.41, 4559.30, 1418.51, 1406.62, and 927.09, as well as the MAEs of 1186.11, 4556.12, 3614.79, 1119.15, 1047.78, and 625.36 indicate that the prediction errors are at a tolerable level. It can be also revealed by the MAPEs of 4.3821, 17.4724, 15.7898, 4.2317, 3.6884, and 2.3082%.

### Comparative assessment

The idea of evaluating some benchmark methods is a well-known way of demonstrating the efficiency of a new method. In this work, the performance of the proposed SBO was compared with five capable metaheuristic techniques, namely the BSA, EWA, SOSA, SSA, and WDO. All results manifested that the SBO is superior to the benchmarks in terms of all accuracy indicators. For example, the smallest relative error (i.e., MAPE) in both training and testing phases were obtained by the SBO to be 2.5060 and 2.3082%, respectively.

For a better evaluation, a scoring system is developed among the models to compare their accuracies. According to earlier literature, using scoring systems is a popular approach for comparison of machine learning models^88^. In this regard, for each accuracy indicator, a score is designated to each model with respect to its rank so that the higher the accuracy, the larger the score. In this research, there are 6 models, and accordingly, the scores may vary from 1 to 6. As an example, the EWA-ANN had the highest RMSE and lowest R; hence, its score is 1 for both accuracy criteria. In contrast, the SBO-ANN had the highest R and lowest RMSE; hence, its score is 6 for both accuracy criteria. For each model, an overall score is calculated (as the summation of all obtained scores) to make the final judgment of ranking in each phase.

The results are shown in Table 3. Apart from the SBO which grasped the largest overall score = 24 in both phases, the SSA and WDO have a close competition for the second position. Their overall scores = 18 in the training phase, while the WDO gave a better testing performance (with overall scores of 20 vs. 16). The BSA emerged as the fourth accurate model, followed by the SOSA and EWA (with respective overall scores of 12, 8, and 4 in both phases).

**Table 3:** Scoring system and obtained accuracy indicators.

Number	Model	Training phase					Testing phase				
		RMSE	MAPE	MAE	R	Overall	RMSE	MAPE	MAE	R	Overall
Obtained indicator	BSA-ANN	1554.91	4.1591	1137.59	0.99485	–	1507.82	4.3821	1186.11	0.99485	–
	EWA-ANN	6408.07	19.9943	5056.13	0.90565	–	5906.41	17.4724	4556.12	0.91217	–
	SOSA-ANN	4653.58	16.0975	3652.30	0.95233	–	4559.30	15.7898	3614.79	0.95068	–
	SSA-ANN	1233.51	3.7931	965.20	0.99663	–	1418.51	4.2317	1119.15	0.99519	–
	WDA-ANN	1247.45	3.4434	947.07	0.99655	–	1406.62	3.6884	1047.78	0.99522	–
	SBO-ANN	934.15	2.5060	669.75	0.99817	–	927.09	2.3082	625.36	0.99802	–
Score	BSA-ANN	3	3	3	3	12	3	3	3	3	12
	EWA-ANN	1	1	1	1	4	1	1	1	1	4
	SOSA-ANN	2	2	2	2	8	2	2	2	2	8
	SSA-ANN	5	4	4	5	18	4	4	4	4	16
	WDA-ANN	4	5	5	4	18	5	5	5	5	20
	SBO-ANN	6	6	6	6	24	6	6	6	6	24

**Figure 7:**
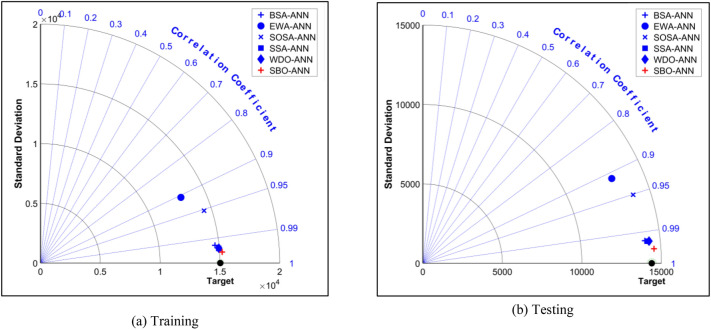
Comparative Taylor Diagrams for graphical comparison.

Moreover, Fig. 7 plots the Taylor Diagrams for graphical comparison. In this figure, the points are positioned with respect to their standard deviation and correlation coefficients simultaneously. The point of the target data is black and its position should be compared to the points of the used models. As is seen, the red plus sign which corresponds to the SBO-ANN model is the nearest to the Target point in both training and testing phases, followed by the points of the BSA-ANN, WDO-ANN, and SSA-ANN. After that, there is a considerable gap between the mentioned points and those of the SOSA-ANN and EWA-ANN; demonstrating poorer predictions for these two models. Altogether, the comparison shown in Fig. 7 is in agreement with Table 3; both declaring the SBO-ANN as the outstanding model of the study.

**Figure 8:**
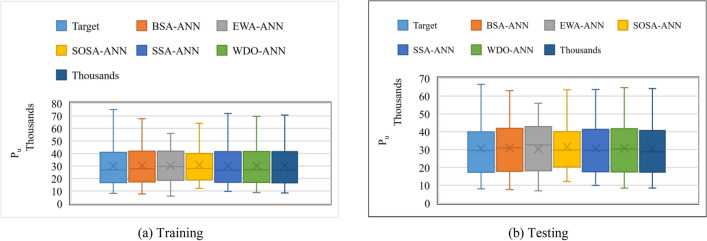
Comparative boxplots of the target and output *P*_*u*_s (In each box, the line and cross mark represent the median and mean values, respectively).

For further comparison, Fig. 8 depicts the boxplots of the target and predicted *P*_*u*_s. Visual interpretation of this figure confirms the comparison results in Fig. 7 and Table 3, because the results of the SBO-ANN are closest to the target values (in terms of minimum, mean, maximum, and median values).

### An explicit formula

**Figure 9:**
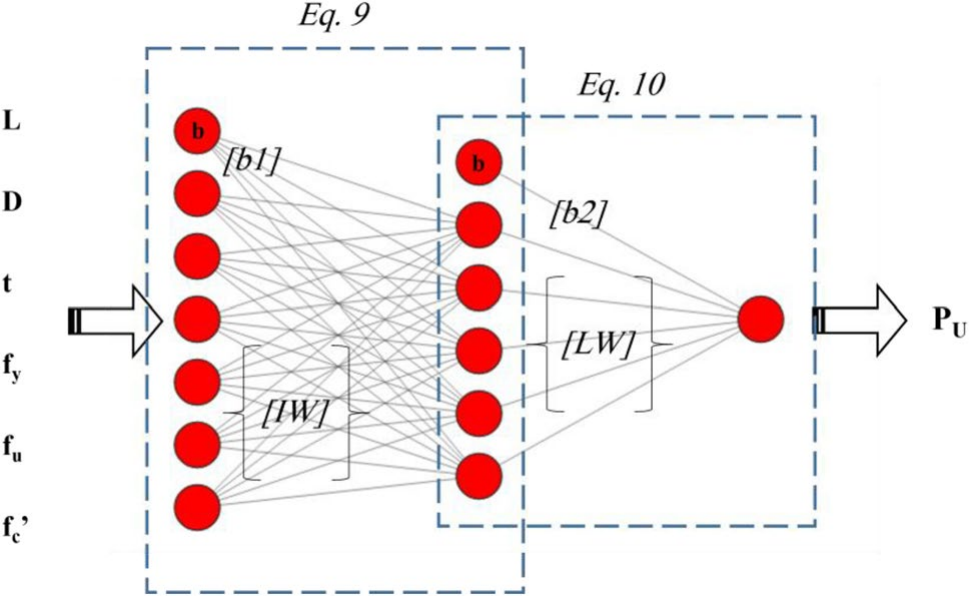
Schematic structure of the used ANN and the components of its equation.

This section is concerned with presenting a neural formula that can predict the *P*_*u*_. All hybrid models used in this work had the same structure of the neural network (i.e., 6 × 5 × 1) as shown in Fig. 9. The difference was their computational weights and biases that were tuned by various metaheuristic algorithms. It was decided to present the formula of the SBO-ANN as it provided a more accurate solution.

In order to extract the formula of a three-layered ANN, two equations should be created (see Fig. 9):

1. One large equation that accounts for the computations in the middle layer as given in Eq. 9:
  9 $$ \left[ Q \right] = \frac{2}{{1 + e^{{ - 2\left( {\left[ {IW} \right] . \left[ {Input} \right]} \right) + \left[ {b1} \right])}} }} - 1,\quad (i = 1,2, \ldots ,5) $$
2. Another equation that accounts for the computations in the output layer; releasing the final *P*_*u*_ as given in Eq. 10:
  10 $$ P_{u} = \left[ {LW} \right] \cdot \left[ Q \right] + \left[ {b2} \right], $$
  in which $[Q]$ is the outcome of the middle layer which is the input of the output layer. Also, *[Input]* is the vector of inputs, *[IW]* is the vector of weights between the input and hidden neurons, *[b1]* is the vector of biases of the hidden neurons, *[LW]* is the vector of weights between the output and hidden neurons, and *[b2]* is the bias of the output neuron; as introduced below:
  11 $$\left[Input\right]=\left[\begin{array}{c}D\\ L\\ t\\ {f}_{y}\\ {f}_{u}\\ {f}_{c}{\prime}\end{array}\right],$$
  12 $$\left[IW\right]= \left[\begin{array}{cccccc}0.7456& -0.9534& -0.8111& -0.8484& 0.3690& 0.6106\\ 0.8780& -0.4792& 1.0182& -0.1693& -1.0045& 0.5260\\ -0.8427& 0.1059& 1.0328& 0.9392& 0.7887& -0.2435\\ 0.9189& 1.0170& -0.0325& 0.6495& 0.9647& 0.3456\\ 0.2949& 1.0359& 0.6691& 1.0239& 0.3983& -0.7327\end{array}\right],$$
  13 $$\left[b1\right]=\left[\begin{array}{c}-1.8307\\ -0.9154\\ 0.0000\\ 0.9154\\ 1.8307\end{array}\right],$$
  14 $$LW=\left[\begin{array}{ccccc}0.4121& -0.9363& -0.4462& -0.9077& -0.8057\end{array}\right],$$
  15 $$b2= \left[\begin{array}{c}0.6469\end{array}\right],$$

### Discussion, limitations, and future work

As is known, preventing computational drawbacks such as overfitting and local minima is of great importance in machine learning implementations. In this work, this issue was taken under control using powerful optimization algorithms that employ specific strategies to keep their solution safe from computational weaknesses. Therefore, it can be said that the used ANNs have not experienced overfitting and local minima problems.

In comparison with solutions that were suggested in earlier studies, it can be said that the proposed SBO-ANN achieved significant improvements. In a study by Zheng et al.^89^, three optimization algorithms of equilibrium optimization (EO)^90^, grey wolf optimization (GWO)^91^, and Harris hawk optimizer (HHO)^92^ were combined with ANFIS^93^ for the *P*_*u*_ prediction. Likewise, two ANNs were optimized by Hu et al.^94^ using social ski-driver (SSD)^95^ and future search algorithm (FSA)^96^. Table 4 compares the RMSE, MAPE, MAE, and R values of these models with the SBO-ANN. According to these results, the accuracy of the SBO-ANN model is higher than all five benchmarks, due to lower error values (RMSE, MAPE, MAE) and higher R values in both training and phases.

**Table 4:** Comparative accuracy indicators with earlier literature.

Study	Model	Training phase				Testing phase			
		RMSE	MAPE	MAE	R	RMSE	MAPE	MAE	R
^89^	EO-ANFIS	1275.95	4.0485	956.77	0.99640	1346.17	4.1809	1022.85	0.99564
	GWO-ANFIS	2944.91	10.3734	2291.41	0.98062	2872.76	10.5947	2324.19	0.98006
	HHO-ANFIS	1422.89	4.5521	1071.82	0.99551	1492.50	4.8947	1184.65	0.99462
^94^	FSA-ANN	2215.30	6.22	1673.64	0.98909	2136.70	6.10	1662.89	0.98896
	SSD-ANN	2614.68	7.95	1972.00	0.98522	2604.59	8.37	2016.41	0.98435
This study	SBO-ANN	934.15	2.5060	669.75	0.99817	927.09	2.3082	625.36	0.99802

Referring to Figs. 4 and 5, one may argue that while all models achieve a reliable R (> 0.90), there are notable differences between the obtained values. For instance, R_EWA-ANN_ = 0.90565 vs. R_SBO-ANN_ = 0.99817 in the training phase and R_EWA-ANN_ = 0.91217 vs. R_SBO-ANN_ = 0.99802 in the testing phase. Since all models have been trained and tested using the same datasets, the reason behind these differences must be sought in the optimization ability of the used algorithms (see Fig. 3). On the other hand, based on Table 3, it should be noted that there is a consistency between the training and testing performance of the models; as the model with the strongest training yielded the best testing quality and vice versa.

**Figure 10:**
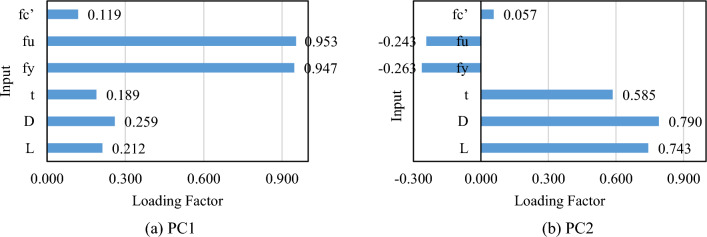
The PCA results for identifying the most significant inputs.

In machine learning applications, it is essential to understand the significance of the used input factors. Statistical analysis is commonly used for this purpose to see which input factors have the greatest effect on the prediction of a given target parameter (here *P*_*u*_). In this work, principal component analysis (PCA)^97^ is used to establish an importance assessment method. In the PCA method, after analyzing the dataset:

1. The primary outcomes are several components each having an eigenvalue. As a well-accepted threshold, eigenvalue = 1 is used to determine which components are considered principal (if eigenvalue > 1). In this work, among the six created components, two of them reached an eigenvalue > 1. These two components are called PC1 and PC2 which together account for nearly 60.30% of variation in data.
2. PC1 and PC2 are then analyzed to identify the most significant inputs. Each input factor in these PCs is attributed to a loading factor. In case the loading factor is > 0.75 (or < -0.75), the input is considered significant^98^. Figure 10 shows the results, according to which, *f*_*y*_ and *f*_*u*_ in PC1 along with *D* in PC2 satisfy this condition.

Considering the limitations of this study, a number of ideas can be raised for future efforts as follows:

1. Replacing the used metaheuristic algorithms with newer members of this family and comparing the results toward improving the obtained solution.
2. Exposing the models to external datasets in order to extend their generalizability.
3. Taking advantage of the PCA results in order to train the models using the most important input factors and compare them with the models trained by the original dataset.
4. Developing a graphical user interface (GUI) from the suggested models.

## Conclusions

This paper offered a novel hybrid algorithm for approximating the axial compression capacity of concrete-filled steel tube columns. To this end, an ANN was properly supervised by the satin bowerbird optimizer to analyze the dependency of the *P*_*u*_ on several input parameters. To achieve the optimum configuration of the model, the best population size of the SBO was determined. The goodness of the training results reflected a high learning accuracy of the suggested model (e.g., MAPE = 2.5060). This model could also predict the *P*_*u*_ for unseen samples with low error (e.g., MAPE = 2.3082). In both phases, the SBO-ANN surpassed five other metaheuristic ensembles, namely BSA-ANN, EWA-ANN, SOSA-ANN, SSA-ANN, and WDA-ANN. In addition, the proposed model presented more accurate results compared to several methods from the literature. Moreover, the results of principal component analysis revealed that *f*_*y*_, *f*_*u*_, and *D* are the most important parameters on the *P*_*u*_. Altogether, the findings of this research can be practically used for optimizing the CFSTC design. Finally, an explicit formula was derived from the developed model which can predict the *P*_*u*_ without the need for computer-aided software. Regarding the limitations, some ideas were suggested for future efforts toward optimizing the model and data leading to better solutions.

## Data Availability

The data analysed during this study are taken from an earlier study Ref.^70^ and are publicly available in the mentioned paper.
